# New Indications for Hematopoietic Stem Cell Gene Therapy in Lysosomal Storage Disorders

**DOI:** 10.3389/fonc.2022.885639

**Published:** 2022-05-13

**Authors:** Linda Rossini, Caterina Durante, Antonio Marzollo, Alessandra Biffi

**Affiliations:** ^1^Pediatric Hematology, Oncology and Stem Cell Transplant Division, Padua University Hospital, Padua, Italy; ^2^Fondazione Citta’ della Speranza, Istituto di Ricerca Pediatrica, Padua, Italy; ^3^Maternal and Child Health Department, Padua University, Padua, Italy

**Keywords:** lysosomal storage disorders (LSD), mucopolisacaridosis, Hunter disease, neuronal ceroid lipofucinosis, Batten disease, gene therapy (GT), hematopoietic stem cell (HSC)

## Abstract

Lysosomal storage disorders (LSDs) are a heterogenous group of disorders due to genetically determined deficits of lysosomal enzymes. The specific molecular mechanism and disease phenotype depends on the type of storage material. Several disorders affect the brain resulting in severe clinical manifestations that substantially impact the expectancy and quality of life. Current treatment modalities for LSDs include enzyme replacement therapy (ERT) and hematopoietic cell transplantation (HCT) from allogeneic healthy donors, but are available for a limited number of disorders and lack efficacy on several clinical manifestations. Hematopoietic stem cell gene therapy (HSC GT) based on integrating lentiviral vectors resulted in robust clinical benefit when administered to patients affected by Metachromatic Leukodystrophy, for whom it is now available as a registered medicinal product. More recently, HSC GT has also shown promising results in Hurler syndrome patients. Here, we discuss possible novel HSC GT indications that are currently under development. If these novel drugs will prove effective, they might represent a new standard of care for these disorders, but several challenges will need to be addresses, including defining and possibly expanding the patient population for whom HSC GT could be efficacious.

## Introduction

Lysosomal storage disorders (LSD) are genetically determined inborn errors of metabolism (IEM) that occur when pathogenic variants affect genes codifying for proteins involved in lysosomal function. Depending on the involved metabolic pathway, affected lysosomes become unable to digest proteins, nucleic acids, carbohydrates or lipids, and this results in the accumulation of undegraded metabolites, leading to cytotoxicity (1). These disorders are generally classified based on the specific enzymatic deficiency and accumulated metabolite, and include Mucopolysaccharidoses (MPSs), Oligosaccharidoses, Sphingolipidoses and Neuronal Ceroid Lipofuscinoses (NCLs). Clinical manifestations depend on the specific molecule(s) that cannot be degraded and are very diverse among different LSDs, affecting several different organs and tissues. In a large subset of disorders, based on the tissue distribution of the storage material, the central nervous system (CNS) is particularly exposed to severe damage, with a wide range of neurological manifestations but generally with a progressively degenerative evolution. Severe neuroinflammation, linked to metabolite accumulation in glia and neural cells and their reactive activation, is a consistent finding in animal models and may be a factor implicated in the progression of neurodegeneration (2). Treatment options for LSDs are very limited and available for few disorders. Enzyme replacement therapy (ERT), namely the administration of a recombinant form of the lacking enzyme, is only available for few disorders and has no efficacy on the neurological manifestations of those LSDs, since the intrinsic properties of these drugs impede the crossing of the Blood Brain Barrier (BBB) (3, 4).

## Hematopoietic Cell Transplantation for LSDs

Hematopoietic Cell Transplantation (HCT) from compatible, allogeneic, healthy donors was originally proposed to treat patients with LSDs due to the ability of the transplanted hematopoietic cells and of their mature myeloid progeny to become a stable source of the defective enzyme for the affected tissues, including the CNS. The enzyme provided by healthy cells can be excreted in the intracellular matrix and taken up by non-hematopoietic cells, resulting in cross correction of the metabolic defect in other cellular populations (5). Despite this sound rational, however, the efficacy of HCT was proven only in few selected LSDs, and clinical situations, and is limited by several factors. Firstly, to benefit from HCT, patients are required to have pre-symptomatic or very early symptomatic disease. This is due to the delay of CNS engraftment, since therapeutically sufficient donor cell contribution to CNS myeloid population might require 12 to 18 months post-transplant to occur (6). Thus, most of the patients diagnosed based on clinical manifestations, possibly after a long diagnostic odyssey due to the rarity of the disease, might have irreversible neurological damage, too severe to benefit from HCT. For example, patients with Early Infantile Globoid Cell Leukodystrophy (GLD, Krabbe disease) benefit from transplant only if it is performed well before the onset of symptoms, ideally in the first 3 months of life, or even within the first 30 days of life for most severe cases (6). Similarly, in patients with Hurler syndrome, variable but substantial residual disease burden is seen in the long term, with improved cognitive development in patients of younger age at time of transplantation (7). Secondly, the efficacy of HCT might be limited to specific organs and clinical manifestation. For instance, skeletal deformities in patients with MPS I are generally mitigated but not completely abrogated by HCT (8). This is probably due to the inability of the corrected cells to reach sufficient engraftment in the already constituted bone niche. Thus, patients often require life-long treatments and surgical interventions to correct these deformities despite a successful HCT. Thirdly, HCT has an established indication only for patients affected by Hurler syndrome (severe MPS I) if younger than 2.5 years old (7), Krabbe Disease, Metachromatic Leukodystrophy (MLD) in very selected cases (9), and selected patients with MPS II (10). Data regarding other disorders are largely anecdotal and the risk/benefit ratio is not clearly defined. For instance, most patients with MLD ultimately experience neurologic decline following HCT, and only patients transplanted in pre-symptomatic stage have shown some benefit from the procedure (9). Lastly, the use of HCT is limited by the availability of compatible donors, as potentially HLA-matched siblings or family members are often carriers of the disease and not suitable as donors. Other risks specifically associated with HCT include transplant associated toxicity, short-term and long-term adverse effects of chemotherapy given as conditioning, graft failure, infections, and Graft versus Host Disease (GvHD). Moreover, the conditioning regimen and the requirement for long hospitalization and isolation might negatively impact the neurological function of patients (6).

## Current Hematopoietic Stem Cell Gene Therapy Approaches for LSDs

In this perspective, *ex vivo* autologous hematopoietic stem cell (HSC) based gene therapy (GT) has the potential to provide an effective and safe strategy to correct the genetic defect in patients affected by LSDs. HSCs collected from the patients can be efficiently transduced and corrected in their metabolic defect by integrating viral vectors, such as lentiviral vectors (LVs), *ex vivo* to constitutively express the functional enzyme, hence possibly generating a sustained and potentially lifelong source of therapeutic enzyme upon transplantation (11). The mechanism of action is similar to allogenic HSCT, but genetically modified autologous HSCs are employed instead of allogenic HSCs to engraft and differentiate into myeloid and lymphoid cells that would repopulate hematopoietic and not-hematopoietic organs of recipients upon transplantation (12). The GT approach has several advantages over allogeneic HCT for LSDs. First, cells are collected from the patient and thus the risk of GvHD is absent, allowing to avoid the use of immunosuppressive drugs post-transplant and favoring early immune reconstitution, lowering the risk of opportunistic infections. Secondly, the use of LVs for gene addition allows enhancing the level of enzyme production by the transplanted cells and their progeny up to supraphysiological levels that may allow a more efficient cross-correction and possibly a greater clinical outcome as compared to allogenic HCT (13).

This approach for LSDs was first successfully applied to patients affected by MLD, a prototypical LSD due to arylsulfatase A deficiency and characterized by unrelenting severe demyelination and secondary neurodegeneration. Treatment of pre-symptomatic late infantile and pre-symptomatic or very early symptomatic early juvenile patients resulted in prevention of disease onset or halted disease progression, as compared with historical untreated control patients (14–16). Based on the results from treatment of a large patients’ cohort, this approach is currently available in Europe as an approved drug with the trade name Libmedly^®^. In patients with Hurler syndrome, HSC GT has been recently investigated in a Phase I/II clinical trial for patients lacking a suitable donor. Results from the eight patients enrolled in the study demonstrated stable cognitive performance, continued motor development, improved or stable findings at magnetic resonance imaging of the brain and spine, reduced joint stiffness, and normal statural growth (17). For both these indications, patients were given a fully myeloablative conditioning regimen based on the alkylating agent Busulfan, which is also part of the standard regimen for HCT in LSDs. Despite resulting in some toxicity, the use of this protocol is required to allow sufficient engraftment of the transplanted cells in the hematopoietic system and also in the CNS, as demonstrated by animal models (18).

The most relevant potential risk of GT over HCT is related to vector-mediated insertional oncogenesis. This is due to the possibility that the insertion site of viral genome would lie close to an oncogene, resulting in an alteration of the expression patterns, a survival advantage of the affected cells and the development of myelodysplasia or leukemia (19). These events are strictly dependent on the vector employed for gene transfer and to the disease context, but have never been reported in patients treated with 3^rd^ generation LVs with eukaryotic internal promoters over a broad range of clinical applications.

Adeno associated vectors (AAV) have also been tested in the setting of *in vivo* GT applications in preclinical models of several neurometabolic diseases with some success (20, 21). In most of the cases, the *in vivo* administration of the recombinant AAVs was performed systemically or directly in the CNS (either in the cerebrospinal fluid (CSF) or through intraparenchymal injections) (22–30). The clinical translation of promising preclinical results however, failed to demonstrate a similar extent of benefit, likely because of limited biodistribution and/or lack of adequate therapeutic gene expression in key target cells. Moreover, the invasiveness of the intra-CNS delivery approach and the risk of immune responses that may be triggered by the AAVs could limit their application (31).

In the near future, several other challenges will need to be addressed to allow a widespread use of HSC GT. Firstly, the need for proper identification of candidate patients, ideally by newborn screening programs and up-to-date phenotype prediction based on genotype. Moreover, centralized production and high costs of the medicinal product as currently manufactured may limit the availability of HSC GT to resource-poor settings. Thus, novel technologies might be needed to allow the widespread use of HSC GT over a wide range of indications, reduce the costs of the drug and the burden of drug development.

## Novel Indications for Hematopoietic Stem Cell Gene Therapy

### Mucopolysaccharidosis Type II

Mucopolysaccharidosis type II (MPS II, Hunter syndrome), is a LSD that affects 0.30-0.71 every 100,000 live births worldwide (32). It is caused by the genetic deficiency of the enzyme iduronate-2-sulfatase (IDS), due to a deleterious variant in the *IDS* gene. IDS deficiency causes the pathological accumulation of dermatan and heparan sulphate (DS and HS) within lysosomes of virtually all type of cells in the body (33), resulting in progressive cellular and multi-organ dysfunction. The spectrum of clinical manifestation is wide and clinically patients with MPS II are classified into two phenotypic variants, neuronopathic (severe) or non-neuronopathic (attenuated), based on the presence or absence of early onset, progressive cognitive deterioration (34, 35) (**Table 1**). Disease progression is faster in patients with the severe phenotype, with early death usually in the mid-teenage years, whereas patients with the attenuated phenotype may live until adulthood (36).

**Table 1:** Clinical manifestation and available treatments for selected LSDs.

	MPS II/Hunter syndrome	MPS IIIA/Sanfilippo syndrome A	MPS IIIB/Sanfilippo syndrome B	CLN 1 disease
**OMIM#**	309900	252900	252920	256730
**ORPHA#**	580	581	581	216
**Enzyme**	iduronate-2-sulfatase	N-sulfoglucosamine sulfohydrolase	N-acetyl-α- glucosaminidase	palmitoyl-protein thioesterase-1
**Inheritance**	XLR	AR	AR	AR
**Gene/Locus**	*IDS*/Xq28	*SGSH*/17q25.3	*NAGLU*/17q21.2	*PPT1*/1p34
**Clinical manifestations**			Similar to MPS-IIIA but with prominently neurological manifestations	Different time and order of onset in the infantil, late infantil, juvenile and adult form
*Muscoskeletal*	Hip dysplasia, dysostosis, carpal tunnel syndrome, arthropathy, joint contractures	Joint stiffness, dysostosis multiplex, skoliosis, kiphosis, misshaped or hypoplastic vertebral bodies, hip deformities, osteonecrosis of the femoral head, low bone mass	same as MPS-IIIA	no
*Respiratory*	Recurrent upper respiratory tract infections	Recurrent upper respiratory tract infections	same as MPS-IIIA	no
*Cardiac*	Valvular thickening	Valvular anomalies	same as MPS-IIIA	no
*Gastrointestinal*	Hepatosplenomegaly, lax abdominal muscles with consequent abdominal distension	Hepatosplenomegaly, chronic or recurrent loose stools and/or constipation, dysphagia	same as MPS-IIIA	no
*Neurological*	Seizures, cervical mielopathy	Language and motor delay/regression, intellectual disability, seizures, diffused cerebral atrophy, spinal stenosis or compressions, spasticity	same as MPS-IIIA	Hypotonia, psychomotor regression, myoclonus, seizures, visual loss, ataxia, spasticity, parkinsonism acquired microcephaly
*Neuropsychological*	Speech delay, hyperactivity, aggressiveness, intellectual disability	Behavioural problems, sleep disturbances, autism spectrum disorder	same as MPS-IIIA	Loss of interaction, depression
*Dermatological*	Papular pearly rash across the scapulae, Mongolian blue spots	Thick skin, thick hair and hirsutism	same as MPS-IIIA	no
*ORL*	Recurrent otitis media, sleep obstructive apnoea, hearing loss	Recurrent otitis media, sleep obstructive apnoea, hearing loss	same as MPS-IIIA	no
*Ophtalmic*	Papilledema without raised intracranial pressure, retinal degeneration	Corneal opacities, optic nerve atrophy, retinal degeneration	same as MPS-IIIA	no
*Others*	Umbelical and inguinal hernias	Umbelical and inguinal hernias	same as MPS-IIIA	no
*Appearance*	Short in statures, pletoric, coarse face with thickened alae nasi, lips, ear lobules and tongue, macrocephaly, hypertrichosis	Coarse face, dolicocephaly or macrocephaly, thickened alae nasi, lips, ear helices or lobules, macroglossia.	same as MPS-IIIA	no
**Life expectancy (untreated)**	Early death in mid-teenage years; pts with attenuated phenotype may live until adulthood	Death usually occurs in the 2nd or 3rd decade	same as MPS-IIIA	8-14 y in the infantile and late infantile forms
**ERT availability**	Elaprase®	in development	in development	none
**ERT issues**	Weekly infusions; limited therapeutic effect on tracheal deformities, join stiffness, bone deformities and hearing loss; insufficient CNS penetrance	no effect on CNS manifestations, despite IT administration	trial ongoing	N/A
**HCT indications and issues**	Limited benefit, risk for morbidity and mortality	No benefit	No benefit	No benefit
**Ongoing AAV GT trials**	NCT03566043, NCT04571970	NCT04088734, NCT02716246	NCT03315182	
**Ongoing HSC GT trials**		NCT04201405		

AR, autosomal recessive; XL, X-linked.

Elaprase^®^ is a recombinant human IDS enzyme that can be administered intravenously as ERT in MPS II children. It is the only medicinal product approved in various EU countries for the treatment of MPS II. Elaprase® weekly use results in a positive treatment effect with reduction of GAG levels in urine and of liver and spleen volume. However, no univocal evidence exists of efficacy of Elaprase® in impacting the progression of pulmonary and cardiological disease manifestations, nor of neurologic/cognitive abnormalities (37–39), limiting its use in patients with severe cognitive symptoms (34). Efforts have been made to favor ERT delivery across the BBB and improve the neurological outcome. Among others, an open label phase 2/3 clinical trial of Pabinafusp Alfa (IDS fused with anti-human transferrin receptor antibody, exploiting transferrin receptor-mediated transcytosis through the BBB) showed decrease in HS CSF levels, associated to lack of progression or positive changes in the neurodevelopmental evaluations of the treated patients (40). Wider and more durable studies are needed to confirm these data.

Additionally, ERT requires lifelong weekly infusions, which can take hours, creating a major burden for patients and their caregivers, and induce the formation of neutralizing antibodies (32). As an alternative to Elaprase®, HCT using umbilical cord blood, peripheral blood HSCs or bone marrow has been tested in limited subsets of patients with variable outcomes (10, 41). In particular, a recent review of 119 retrospective, published cases plus 27 newly described patients reported some improvement/no progression of abnormal MRI findings as well as some clinical benefit, in terms of amelioration of somatic features, joint movements and activities of daily living, following HCT. Despite the work concludes in favor of HCT as compared to ERT in the treatment of MPS II, no controlled clinical studies have been conducted to date evaluating the effects of HCT in patients with MPS II and for this reason HCT is not considered as the standard of care for MPS II and its use is limited to selected patients with early-symptomatic disease that do not tolerate ERT.

Multiple HSC GT approaches have been proposed for the treatment of MPS II. HSC GT with a second- generation LV carrying a codon optimized human *IDS* gene has shown improvement in lysosomal storage and autophagic dysfunction in the brain in a mouse model of MPS II but required a strong preconditioning to provide a substantial cognitive function improvement (42, 43). In another study, the *IDS* transgene with the myeloid-specific CD11b promoter was fused to the receptor-binding domain of human apolipoprotein E (LV.IDS.ApoEII) to deliver the therapeutic enzyme more efficiently through the BBB. This approach showed better correction of memory deficits, inflammation, and HS storage in the brain as compared to a LV encoding only *IDS* (44). Recently, another LV encoding the human *IDS* under control of the human phosphoglycerate-kinase (PGK) promoter has been developed and the clinical development of this drug product is planned in the next few years, with a fully myeloablative busulfan conditioning prior to the administration of the genetically modified cells (45).

The first human study on safety and efficacy of the intra-CNS administration of increasing doses of recombinant AAV expressing human IDS (Regenxbio, RGX-121) in patients with MPS II is currently ongoing (NCT03566043). Enrolled patients receive a single intracisternal injection of RGX-121 and immunosuppression therapy for 48 weeks. Interim results published in January 2021 show a good tolerability profile, with biomarker levels and neurodevelopmental function suggesting some potential disease modifying effects (46, 47). The study is still recruiting and is estimated to be completed in 2024.

Several challenges will need to be addressed during the clinical development of a drug based on any of this evidence. Most importantly, the assessments will need to take into consideration the wide spectrum of clinical severity of patients with MPS-II, with some patients developing a neuronopathic form. As is the case of other metabolic disorders such as X-linked Adrenoleukodystrophy, the severity of the CNS manifestations is extremely difficult to predict in MPS II based on genetic and functional data alone (6, 41, 48, 49). Other measures of disease severity, such as clinical severity score (50), neuroimaging findings (e.g., atrophy, enlarged perivascular space, white matter lesions, hydrocephalus) or the presence of MPS II-related CNS symptoms (e.g. behavior problems, hyperactivity) (35) may be indicative of a severe form, but these signs appear only when the CNS damage is already established. Thus, validation of early predictors of severity and of CNS involvement would be of great relevance for this new indication. Importantly, however, as the multisystemic nature of the disease in both neuronopathic and non-neuronopathic forms leads to manifestations that are life-threatening, greatly impact on the quality of life of patients and are only partially controlled by ERT (41, 51, 52), it cannot be excluded that gene therapy in MPS II may be offered to patients in whom CNS involvement could not be predicted with certainty, particularly if they develop neutralizing antibodies to ERT.

### Mucopolysaccharidosis Type IIIA and B

Mucopolysaccharidosis type III (MPS III, Sanfilippo disease), is a group of 4 autosomal recessive LSDs caused by pathogenic variants in *SGSH*, *NAGLU*, *HGSNAT* and *GNS*, respectively. Overall MPS III has a prevalence of 1:50 000 to 1:250 000, with MPS IIIA and IIIB being the most common forms. Clinical characteristics are similar to MPS II, with MPS IIIB patients showing predominantly CNS manifestations and less severe systemic symptoms (**Table 1**). The first manifestations are usually evident in the first years of life, and the impairment is progressive with death occurring in the 2^nd^ or 3^rd^ decade of life in most of the patients (53, 54). Currently, no specific treatment is available for MPS III patients and only supportive treatment is available (54). HCT is not currently considered as a treatment option for MPS III due to the lack of neurologic benefit, even when performed early in the course of the disease (55). Due to the limited ability of intravenous ERT to pass the BBB and modify the neurological outcome, intravenous ERT has not been pursued in MPS III as intensively as in other LSDs and there is currently no approved product available. Alternative routes of administration such as the intrathecal (IT) one, have been tested, but study endpoints aimed at evaluating efficacy on neurological function were not met (56). Multiple *in vivo* AAV GT approaches have been proposed and designed for MPS IIIA or MPS IIIB, but a cognitive benefit was not shown, at least in patients with MPS IIIB (57). An *ex-vivo* HSC GT approach has also been tested in mouse models of MPS-IIIA and MPS-IIIB, using a lentiviral vector and the CD11b promoter, driving the expression of the therapeutic enzyme (SGSH and NAGLU, respectively). In both disorders, a complete correction of the metabolic defect in the brain and satisfactory cognitive outcomes were demonstrated in mice (58, 59). Interestingly, targeting brain inflammation with different strategies, including steroid administration and modulation of the TLR4 and inflammasome pathway, was also effective in these models in preventing brain damage (58, 60). Currently, a trial using autologous CD34+ cells transduced with a lentiviral vector containing the human *SGSH* enrolling pre- and early symptomatic MPS-IIIA patients is open in Manchester (NCT04201405) and the first treated patient has been reported, in whom a very high enzymatic activity was shown at 6 months after treatment (61).

### Neuronal Ceroid Lipofuscinosis Type 1

Neuronal ceroid lipofuscinoses (NCLs, Batten disease), are a group of inherited progressive neurodegenerative diseases clinically characterized by a decline of mental and other capacities, epilepsy, and vision loss through retinal degeneration, without any non-neurological manifestation. Histopathological examination shows intracellular accumulation of an autofluorescent material, ceroid lipofuscin, in the neuronal cells in the brain and in the retina of NCL patients (62). Recent advances in molecular genetic studies have resulted in identifying the primary genetic defects responsible for most forms of NCL. First by gene (or protein), and secondarily by the age of onset and clinical features (63, 64), to date at least 14 genetic NCL disorders have been reported and are designated as CLN 1 to CLN 14. Biallelic mutations in the *CLN1* gene result in CLN1-disease, which is one of the few NCLs due to defects in a lysosomal enzyme, the Palmitoyl-protein thioesterase 1 (PPT1) enzyme, being thus of potential interest for HSC GT approaches. CLN 1 disease presents in four main phenotypes with varying ages of onset: the infantile CLN1-disease or Santavouri-Haltia disease (which is the most frequent form), Late infantile CLN1-disease, Juvenile CLN1-disease, and Adult CNL1-disease (65, 66). In almost all CLN1-disease forms, patients are initially healthy with a normal developmental profile and develop a degenerative disease after the onset of symptoms resulting in premature death (66–69). The treatment of patients with CNL1-disease is only symptomatic (66). Allogeneic HCT has been employed to treat NCLs, but normal enzyme levels could be reached only in peripheral blood and not in the CNS, resulting in the absence of clinical benefit both in the animal models and in humans (70, 71). Overall, HCT is currently not recommended as a treatment modality for patients with any form of CLN, not even those associated to lysosomal enzyme deficiencies as CLN 1 (72, 73).

The potential of gene therapy to provide benefit to CNL1-disease has been explored both in the preclinical and clinical settings (74). Several preclinical studies have focused on the use of AAV directly administered to the CNS of CNL1-disease animal models. However, a major limitation of AVV-based therapy is the insufficiently widespread delivery of the functional enzyme to the CNS, including brain and spinal cord, ultimately hindering the clinical success of such approach (24). HSC GT based on a lentiviral vector encoding for a codon optimized version of the human *PPT1* cDNA has also been tested in mice (75). The gene corrected HSCs were administered *via* intravenous infusion, which is the standard delivery route applied to similar GT products (14, 15), and by an additional route, consisting of direct administration of the cells into the cerebroventricular space (ICV), which was shown to provide rapid and effective myeloid engraftment the CNS (76). This approach was shown to prevent and correct CLN 1 disease manifestations in the mouse model, with the combined intravenous and ICV delivery resulting in the greatest extent of benefit also on symptomatic animals (75). Based on these preclinical data, it may be hypothesized that patients with classic infantile and late infantile forms of CLN1, who are known to experience an aggressive form of disease and show rapid deterioration of cognitive faculties (64), could benefit from this approach that allows for rapid enzyme reconstitution in the CNS. This is especially important in patients diagnosed due to the onset of symptoms which is common considering that newborn screening is not currently available for CLN 1 disease (77).

## Conclusions

Recent advances in HSC GT for patients with LSDs has shown that this approach provides a cure for previously untreatable conditions such as MLD and might be efficacious also in other indications such as MPS I. Based on these findings, HSC GT is currently evaluated for other LSDs, and several disease-specific challenges are being addressed. The combination of different delivery modalities, including ICV infusion of genetically modified HSCs, might result in an amelioration of the CNS engraftment and a faster clinical benefit, allowing the treatment of patients with more advanced forms of disease.

## Author Contributions

LR and CD reviewed the literature and drafted the first version of the manuscript and the tables. AM and AB reviewed the draft and described the orphan drug development. All authors contributed to the article and approved the submitted version.

## Funding

Fondazione Città della Speranza ONLUS supports AM in the scientific work.

## Conflict of Interest

AB acts as Principal Investigator on sponsored research agreements with Orchard Therapeutics.

The remaining authors declare that the research was conducted in the absence of any commercial or financial relationships that could be construed as a potential conflict of interest.

## Publisher’s Note

All claims expressed in this article are solely those of the authors and do not necessarily represent those of their affiliated organizations, or those of the publisher, the editors and the reviewers. Any product that may be evaluated in this article, or claim that may be made by its manufacturer, is not guaranteed or endorsed by the publisher.
